# Type 1 Diabetes Immunological Tolerance and Immunotherapy

**DOI:** 10.1155/2011/103738

**Published:** 2011-12-04

**Authors:** Aziz Alami Chentoufi, Vincent Geenen, Nick Giannokakis, Abdelaziz Amrani

**Affiliations:** ^1^Immunology Unit, Pathology & Clinical Laboratory Medicine, King Fahad Medical City, Box 59046, Riyadh 11525, Saudi Arabia; ^2^Laboratory of Immunoendocrinology, Institute of Pathology CHU-B23, University of Liege Center of Immunology (CIL), 4000 Liege-Sart Tilman, Belgium; ^3^Department of Pathology and Immunology, Diabetes Institute, Rangos Research Center, University of Pittsburgh School of Medicine, Pittsburgh, PA 15213, USA; ^4^Immunology Division, Department of Pediatric, University of Sherbrook, Sherbrook, QC, Canada J1H 5N4

Type 1 diabetes (T1D) is a debilitating chronic autoimmune disease (AID) for which there is currently no preventive treatment or therapeutic strategies. Patients with T1D are also at higher risk for other autoimmune disease such as celiac disease. In genetically susceptible individuals, T1D is associated with the generation and activation of autoreactive CD4^+^ and CD8^+^ T cells that infiltrate the pancreas and selectively destroy the insulin-producing *β*-cells in the islets. The impairment of T-cell tolerance in T1D has been reported at many levels including abnormal self-antigen presentation in the thymus and periphery, autoreactive T-cell resistance to apoptosis, unbalanced immunoregulatory T-cell function, and deregulation of Th1/Th2/Th17 axes. Despite the identification of T1D-associated autoantigens and their derived CD4^+^ and CD8^+^ T-cell epitopes, numerous antigen-specific immunoregulatory therapies have failed when evaluated for their efficiency in the prevention and treatment of T1D. The development of antigen-specific tolerization approaches to treat or to prevent T1D would bring exceptionally high economic and health benefits. In this special issue, we accepted original research and review articles highlighting the recent advances in T1D-associated immunological tolerance mechanisms and potential immunotherapeutic strategies.

In the first paper of this special issue, A. A. Chentoufi and V. Geenen have addressed the role of thymic self-antigens expression in the control of self-reactive and regulatory T-cells generation. Also, the authors proposed the concept of negative/tolerogenic self-vaccination to modulate diabetogenic autoimmune responses. In the second paper, G. F. Hoyne described the molecular mechanisms that regulate peripheral immune responses that control organ-specific autoimmunity and highlighted the role of a range of E3 ubiquitin ligases and signaling pathways that influence the development of effector T-cell responses and T1D development. The third paper by A. A. Chentoufi et al. highlighted the recent findings and controversies regarding the tolerogenic properties of interleukin-2 (IL-2) mediated through naturally occurring regulatory T cells and discussed the link between the immunomodulatory role of IL-2 and the pathogenesis of T1D.

In the fourth paper of this special issue, the group of A. Amrani eloquently investigated the abnormal functionality of dendritic cells (DCs) in nonobese diabetic (NOD) mice through the expression of IRF4 and IRF8 genes. The results showed an upregulation of IRF4, but not IRF8, expression in CD11c^+^ splenic DCs of NOD as compared to BALB/c mice and correlated with the increased levels of CD4^+^CD8*α* ^−^ DCs suggesting that IRF4 may be involved in abnormal DC functions in diabetes in NOD mice. In the fifth paper, the group of G. A. Passos investigated the transcriptional modulation of immune reactivity genes occurring through thymocytes maturation into peripheral autoreactive T lymphocytes. The transcriptome of thymocytes and peripheral CD3^+^ T lymphocytes from prediabetic or diabetic mice analyzed through microarray hybridizations identified 2,771 differentially expressed genes. The analysis of the transcriptional activity of thymocytes developing into peripheral T cells revealed sequential participation of genes involved in CD4^+^/CD8^+^ T cell differentiation, tolerance induction by regulatory T cells, and apoptosis soon after T-cell activation, while the emergence of T1D coincided with the expression of cytotoxicity and inflammatory response genes by peripheral T lymphocytes. The sixth paper by M. Delmastro and J. Piganelli described the importance of reactive oxygen species/oxidative stress as well as potential for redox modulation in the context of T1D.

In the following selected research article papers, P. Alard and Y. Zhang groups described novel therapeutic approaches that prevent and/or treat T1D in mouse model. Indeed, P. Alard group have proposed to utilize the microorganism ability to induce tolerogenic DCs to abrogate the proinflammatory process and prevent diabetes development. They have shown that LTA-treated DCs produced much more IL-12 than IL-10 and accelerated diabetes development when transferred into NOD mice. In contrast, stimulation of NOD DCs with L. Casei favored the production of IL-10 over IL-12, and their transfer decreased disease incidence in an IL-10-dependent manner. Similarly, Y. Zhang group have shown that oral administration of the CTB-Ins-GFP protein induced tolerance, delayed the development of diabetic symptoms, and suppressed diabetes onset in NOD mice. Furthermore, CTB-Ins-GFP protein increased the numbers of CD4^+^CD25^+^Foxp3^+^ regulatory T cells in peripheral lymph tissues and affected the biological activity of spleen cells.

In the last series of papers, we have selected review papers describing the antigen-based therapeutic approaches. S. Culina et al. have discussed the different antigen formulations that have been considered for T1D treatment/prevention, such as proteins or peptides, either in their native form or modified ad hoc, DNA plasmids, and cell-based agents. Also, they highlighted the differences between mice model and human data that should be taken into account. Critical parameters such as administration route, dosing, and interval remain largely empirical and need to be further dissected. R. Mallone et al. described T-cell recognition of autoantigens in human T1D and their relevance to the clinical perspective. Finally, B. Philips et al. highlighted the preclinical successes and the excitement generated by phase I trials while offering alternative possibilities and new translational avenues that can be explored given the very recent disappointment in leading agents in more advanced clinical trials.

*Aziz Alami Chentoufi* *Vincent Geenen* *Nick Giannokakis* *Abdelaziz Amrani*

